# The Biological Activities of Oleocanthal from a Molecular Perspective

**DOI:** 10.3390/nu10050570

**Published:** 2018-05-06

**Authors:** Kok-Lun Pang, Kok-Yong Chin

**Affiliations:** 1 Biomedical Science Programme, School of Diagnostic and Applied Health Sciences, Faculty of Health Sciences, Universiti Kebangsaan Malaysia, Kuala Lumpur 50300, Malaysia; pangkoklun@gmail.com; 2 Department of Pharmacology, Universiti Kebangsaan Malaysia Medical Centre, Cheras 56000, Malaysia

**Keywords:** Alzheimer’s disease, antioxidant, cancer, inflammation, neuroprotection, oleocanthal, olive

## Abstract

Oleocanthal is a minor constituent of olive oil with strong anti-inflammatory activities. Since the pathogenesis of many chronic diseases involves inflammatory and oxidative components, oleocanthal is a promising agent to prevent these conditions. This review aimed to summarise the current beneficial health effects of oleocanthal and the molecular basis of its biological actions. The anti-inflammatory, antioxidative, antimicrobial, anticancer and neuroprotective activities of oleocanthal have been examined by previous studies. Of these, studies on the anticancer effects have been the most extensive. Oleocanthal was reported to suppress melanoma, breast, liver, and colon cancer cells. Neurological studies focused on the effects of oleocanthal against Alzheimer’s disease. Oleocanthal improved clearance of the amyloid beta protein from neurons and reduced the inflammation of astrocytes. Despite the positive results, validation of the biological effects of oleocanthal in animal disease models is limited and should be emphasized in the future. As a conclusion, oleocanthal may act together with other bioactive compounds in olive oil to achieve its therapeutic potential. The use of oleocanthal alone as a single therapeutic measure awaits validation from future studies.

## 1. Introduction

Olive oil is the main source of dietary fat in the Mediterranean diet. The daily intake of olive oil in Mediterranean populations is estimated to be 30–50 mL [1,2]. A high dietary intake of olive oil is associated with lower incidences of cancer, cardiovascular disease, metabolic diseases, Alzheimer’s disease and osteoporosis [3,4,5,6,7,8]. Olive oil and its phenolic compounds are known to possess biological activities, including antioxidant, anti-inflammation, anticancer and anti-diabetic activities [9,10,11]. Virgin olive oil diet has been demonstrated to reduce oxidative stress and inflammation in human studies [12,13]. Virgin olive oil possesses several biological activities not shared by other vegetable oils. For instance, it was reported that daily intake of extra virgin olive oil for six months improved synaptic integrity and lowered insoluble protein aggregation in a rat model of Alzheimer’s disease [14]. Previous studies have also demonstrated that virgin olive oil protects low-density lipoprotein from oxidation in patients but sunflower oil does not [15]. Furthermore, sunflower oil and corn oil have failed to demonstrate the same antimicrobial activities as those of virgin olive oil [16]. Oleic acid is found abundantly in olive oil; however, the beneficial effects of olive oil may be contributed by the olive’s minor components [17]. More than 200 different chemical compounds are detected in olive oil, including fatty acid, sterols, carotenoids, terpenoids, flavonoids, tocopherols and olive polyphenols [18]. Olive polyphenols are minor secondary metabolites found in olive oil which consist of multiple phenolic structures [18]. Examples of main olive polyphenols are tyrosol, hydroxytyrosol, oleocanthal (OC), oleacein, olive ligstroside and oleuropein [18]. Among these olive polyphenols, OC is getting more scientific attention due to its interesting biological activities, even though it makes up only 10% of the olive’s polyphenols (100–300 mg/kg olive oil) [1,19].

**Figure 1:**
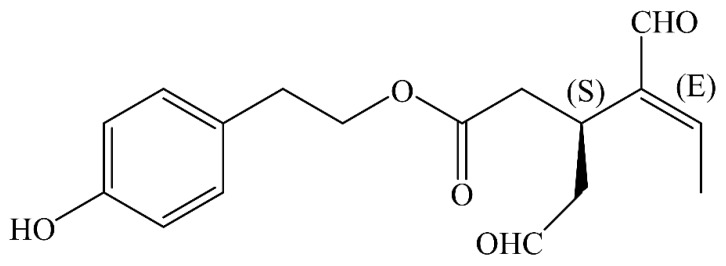
Chemical structure of natural oleocanthal (OC).

OC was first discovered in 1992 [20,21], and its chemical structure was revealed by Montedoro et al. in 1993 [22]. Chemical synthesis of OC has been attempted by several research groups [23,24,25,26,27]. Natural OC exists in the S-configuration of chiral carbon, while synthetic OC is present in the R-configuration [28,29]. The name OC was assigned by Beauchamp et al. (2005), whereby oleo- stands for olive, -canth- for sting sensation, and -al for the presence of aldehyde groups in its chemical structure [28,29]. There are other names for OC, including decarboxymethyl ligstroside aglycone [29], the dialdehydic form of deacetoxy-ligstroside aglycone [28], the dialdehydic form of deacetoxyligstroside glycoside [30], deacetoxy-dialdehydic ligstroside aglycone [31], deacetoxy ligstroside aglycone [32], p-hydroxyphenylethanol-elenolic acid dialdehyde (p-HPEA-DEA or p-HPEA-EDA) [33,34] and tyrosol-elenolic acid dialdehyde (Ty-EDA or Ty-DEA) [35]. The International Union of Pure and Applied Chemistry (IUPAC) name for OC is 2-(4-hydroxyphenyl)ethyl(3S,4E)-4-formyl-3-(2-oxoethyl)hex-4-enoate with the Chemistry Abstracts Service (CAS) number 289030-99-5 [36]. The chemical structure for natural OC is shown in Figure 1. 

The pharmacokinetics and bioavailability of olive polyphenols in humans have been established previously [2,37,38]. However, similar studies on OC are limited. Romero et al. (2007) investigated the stability and diffusion of OC and other olive polyphenols under simulated gastric acid conditions [35]. They showed that most olive polyphenols, including OC, are stable in acidic conditions at 37 °C for up to 4 h [35]. In addition, almost half of the OC diffused from the oil phase into the aqueous solution [16,35]. On the other hand, information on the pharmacokinetics of OC in human is scarce—only one study has found OC and several secoiridoid metabolites in human urine 2–6 h after the ingestion of extra virgin olive oil [32]. OC is mostly metabolized via phase I metabolism, namely hydrogenation, hydroxylation, and hydration. Some of the hydrogenated OC metabolites are further metabolized via phase II metabolism, i.e., glucuronidation [32]. To the best of our knowledge, there is no study being conducted to examine the bioavailability and blood–brain barrier permeability of OC in humans.

## 2. Literature Search

This review aimed to summarise the biological activities of OC. A literature search was performed between 1–28 February 2018 on PubMed and Scopus using the keywords ‘oleocanthal’ OR ‘deacetoxy-ligstroside aglycone’ OR ‘deacetoxy-dialdehydic ligstroside aglycone’ OR ‘p-HPEA-DEA’ OR ‘p-HPEA-DEA’ OR ‘Ty-DEA’ OR ‘Ty-EDA’. We also examined the reference lists of the retrieved articles. Original research articles detailing the health effects of OC published in English were included. Studies of crude olive extract or a mixture of olive polyphenols were excluded from this review. A total of 33 studies were included in the review. 

## 3. Anti-Inflammatory, Antioxidant and Antimicrobial Activities of OC

OC is responsible for the pungency and irritative sensation of virgin olive oil in the throat and oropharyngeal area [23,39]. A molecular study conducted by Peyrot de Gachons et al. (2011) identified that the OC-related pungent sensation was mediated via the activation of transient receptor potential cation channel subtype A1 (TRPA1) [31]. Structureactivity studies further revealed that the OC-mediated TRPA1 activation was greatly dependent on its unsaturated dialdehyde carbonyl moieties [31]. 

OC shares a similar throat irritant sensation with ibuprofen (non-steroidal anti-inflammatory drug, NSAID), although they are structurally distinct [28]. OC inhibits the activities of cyclooxygenase-1 and -2 (COX1 and COX2), the rate-determining enzymes for the synthesis of prostaglandins, which regulate inflammation [28]. The anti-inflammatory properties of OC have been shown to be more potent than ibuprofen [28]. Since TRPA1 activation has been demonstrated to mediate neurogenic and chronic inflammation [40,41,42], the anti-inflammation effect of OC may be related to this receptor. In addition, OC was also shown to significantly abrogate lipopolysaccharide (LPS)-mediated upregulation of COX2 mRNA with 96% inhibition in human isolated normal monocytes [43]. Further, OC inhibited human recombinant 5-lipoxygenase (5-LOX) activity to 52.2 ± 0.6% at a concentration of 10 µM [44], but it had no effect on 15-lipoxygenase (15-LOX) activity [28]. 5-LOX is responsible for the biosynthesis of proinflammatory leukotrienes [45,46]. Adverse drug reactions related to NSAIDs are partly caused by their failure to inhibit 5-LOX [47,48,49]. Therefore, OC serves as a better NSAID candidate by inhibiting COX1/2 and 5-LOX concurrently. In addition, pretreatment with a nontoxic concentration of OC also inhibited LPS-mediated upregulation of inducible nitric oxide synthase (iNOS) and nitric oxide (NO) production in murine chondrogenic ATDC-5 cells and mouse macrophage J774A.1 cells [50,51]. Furthermore, OC also inhibited LPS-mediated upregulation of proinflammatory signalling molecules, including interleukin-1β (IL-1β), interleukin-6 (IL-6), macrophage inflammatory protein-1α (MIP-1α), tumour necrosis factor-α (TNF-α), and granulocyte-macrophage-colony-stimulating factor (GM-CSF) [50]. 

An animal study demonstrated that OC reduced the inflammation activation of astrocytes in the hippocampus of TgSwDI mice, indicated by decreased IL-1β and glial fibrillary acidic protein (GFAP) [52]. In addition, the anti-inflammatory activities of OC (10 and 30 mg/kg, twice a day; intraperitoneal (i.p.) injection for three consecutive days post-traumatic period) also protected the brain cortexes of Wistar albino rats from traumatic degenerative changes and neuronal loss during traumatic brain injury (TBI) [53]. OC also suppressed the inflammatory responses by reducing the endothelial nitric oxide synthase (eNOS) and iNOS expression in TBI rats [53]. OC also reduced TBI-mediated apoptosis and gliosis and partly recovered the injured brain area by inducing the proliferation of angiogenesis and endothelial cells with the upregulation of vascular endothelial growth factor (VEGF) [53].

The antimicrobial activities of olive oil and its polyphenols have been well-documented [54,55,56,57]. A study by Medina et al. (2006) reported a significant association between OC levels and the antimicrobial activity of virgin olive oil against *Escherichia coli* and *Salmonella enterica* [16]. In addition, OC (79.3 µM) also exerted significant antimicrobial activity against *Listeria monocytogenes* [16]. In addition, Romero et al. (2007) reported that OC in concentrations as low as 26 µM could completely eliminate the growth of *Helicobacter pylori* LMG 19449 strains after 60 min of contact [35]. Other olive polyphenols, including tyrosol, hydroxytyrosol, oleuropein aglycon and ligstroside aglycon, were not effective against *Helicobacter pylori* [35]. The antimicrobial activity of oleacein on *Helicobacter pylori* was weaker compared to OC [35]. Moreover, OC was also effective against Gram-positive bacteria *Staphylococcus aureus* CECT 239 strains and *Enterococcus faecalis* CECT 481 strains, but less effective against Gram-negative bacteria *Pseudomonas fluorescens* CECT 378 strains and *Escherichia coli* CECT 434 strains [58]. In parallel with the observation of Romero et al. (2007), OC and oleacein were more potent than tyrosol and hydroxytyrosol, indicating the importance of the dialdehyde moiety in antimicrobial activities [58].

**Figure 2:**
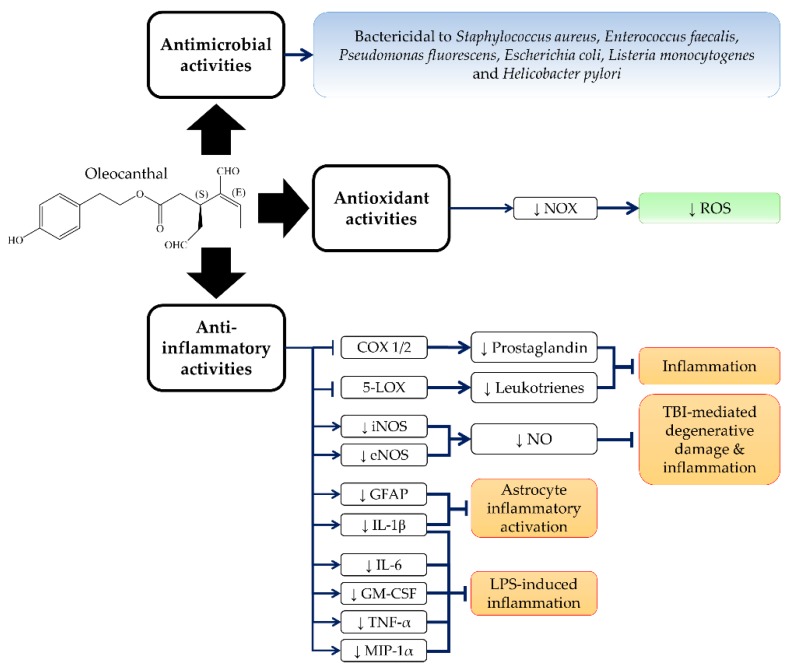
Anti-inflammatory, antioxidant and antimicrobial activities of OC. Abbreviation: ↓ stands for downregulation; NOX = nicotinamide adenine dinucleotide phosphate oxidase; ROS = reactive oxygen species; COX 1/2 = cyclooxygenase 1/2; 5-LOX = 5-lipoxygenase; iNOS = inducible nitric oxide synthase; eNOS = endothelial nitric oxide synthase; NO = nitric oxide; GFAP = glial fibrillary acidic protein; IL-1β = interleukin-1β; IL-6 = interleukin-6; GM-CSF = granulocyte-macrophage colony-stimulating factor; MIP-1α = macrophage inflammatory protein-1α; TBI = traumatic brain injury; LPS = lipopolysaccharide;TNF-α = tumour necrosis factor-α

Antioxidant activities of olive oil and its several olive polyphenols have been reported previously [9,59,60]. However, studies on the antioxidant activities of OC are limited. OC (100 µM) was demonstrated to inhibit nicotinamide adenine dinucleotide phosphate oxidase (NOX) activity and further reduced the intracellular superoxide anion level (36% reduction as compared to control) in isolated human monocytes [10]. A study by Galvano et al. (2007) documented the total amount of olive phenolic compounds, including OC, and the antioxidant capacity of extra virgin olive oil [61]. However, the authors did not investigate the correlation between the OC level and the antioxidant capacity of extra virgin olive oil [61]. Further studies are required to confirm the antioxidant properties of OC. The anti-inflammatory, antioxidant and antimicrobial activities of OC are summarized in Figure 2. 

## 4. Anticancer Properties of OC

Chronic inflammation serves as a promoting factor in the early stage of carcinogenesis, especially in colorectal cancer [62,63]. Therefore, OC may serve as a food chemopreventive candidate due to its promising anti-inflammatory properties. In addition, OC has demonstrated some promising anticancer activities on several cancerous cell lines originating from melanoma [64,65], breast cancer [66,67,68,69,70,71], prostate cancer [66], hepatocellular carcinoma [72,73], colon cancer [73,74], multiple myeloma [75] and leukaemia [76]. The action of OC is selective to cancerous cells and it has less or no cytotoxic effects on primary or non-tumorigenic cell lines, including human adult dermal fibroblast HDFa cells [64], human mammary epithelial MCF10A cells [70], human liver LO2 cells [72], murine macrophages J774A.1 cells [50], human fibroblast BJ cells [77], rat fibroblast 3Y1 cells [77], human lung fibroblast IMR90 cells [77], and isolated primary human hepatocytes [73]. OC induces cytostatic but not cytotoxic effects on BJ cells, 3Y1 cells, and IMR90 cells by inhibiting retinoblastoma protein phosphorylation [77]. The cell proliferation of BJ cells was restored after 72 h of OC treatment [77]. In addition, a high concentration of OC (25 µM) was shown to be cytotoxic to human chondrogenic ATDC-5 cells with p38 activation [51]. However, the relationship between the OC-mediated ATDC-5 cells’ cytotoxicity and p38 signalling remains unknown [51]. In addition, the anticancer activities of OC have been demonstrated in animal studies. OC inhibited cancer cell xenograft tumour growth and metastasis without producing any significant toxic effects on athymic nude mice [65,70,78]. The anticancer properties of OC in various studied models are summarized in Table 1. 

### 4.1. Melanoma

OC has been shown to be cytotoxic to several human melanoma cell lines, including A375 cells, 501Mel cells and G361 cells [64,65]. In addition, OC (20 and 40 µM) was shown to inhibit the colony-forming capacity of A375 cells [65]. A non-cytotoxic concentration of OC (10 µM) was demonstrated to inhibit migration and invasion of human skin malignant melanoma A375 and A2058 cells, possibly via downregulation of matrix metalloproteinase-2/9 (MMP-2/9) [65]. OC also exerted antiangiogenic activity by inhibiting the migration, invasion and tube formation of human umbilical vascular endothelial cells (HUVEC) [65]. Molecular analysis revealed that OC induced melanoma A375 cell apoptosis by activating caspase-9/-3 activation and cleaving poly (ADP-ribose) polymerase (PARP) [65]. OC also downregulated B cell lymphoma-2 (Bcl-2) mRNA and inhibited protein Akt and extracellular signal-regulated kinase-1/2 (ERK 1/2) phosphorylation, thereby inducing the apoptosis of melanoma cells [64]. Moreover, OC inhibited the proliferation and neoplastic transformation of 12-O-tetradecanoylphorbol-13-acetate (TPA)-induced normal mouse epidermis JB6 CI41 cells by blocking ERK 1/2 signalling [74]. 

Furthermore, OC suppressed signal transducer and activator of transcription-3 (STAT3) signalling on A375 cells [65]. OC also interfered STAT3 signalling via suppression of STAT3 phosphorylation, nuclear translocation and transcriptional activity [65]. Subsequently, OC downregulated STAT3 downstream signalling proteins, including myeloid cell leukaemia-1 (Mcl-1), B cell lymphoma-_XL_ (Bcl-_XL_), MMP-2/9 and VEGF, which led to the apoptosis of melanoma cells [65]. OC also inhibited the STAT3 upstream activator, Janus kinase 2 (JAK2), and Src kinase signalling in melanoma cell apoptosis [65]. In female BALB/c athymic nude mice, OC (10 mg/kg/day) inhibited A375 xenograft-induced tumour growth, proliferation and angiogenesis via the activation of caspase-3/9 and the suppression of STAT3, JAK2 and Src kinase signalling [65]. OC (15 mg/kg/day) also significantly reduced lung metastasis of nude mice that received an A375 xenograft tail vein injection [65]. 

### 4.2. Breast Cancer

OC prohibited migratory and invasive activity in MDA-MB-231 by inactivating the breast tumour kinase (Brk)/Paxillin/Rac1 signalling pathway [66,67,68,69,70]. OC suppressed the invasion of breast cancer cell lines by reducing the epithelial-to-mesenchymal transition (EMT), indicated by increased E-cadherin and zona occludens 1 (epithelial markers) and decreased vimentin and β-catenin (mesenchymal markers) upon treatment [70]. OC also demonstrated antiangiogenic activity by downregulating the expression of the microvessel density marker, CD31, in endothelial colony forming cells [66]. In addition, OC also induced G1 arrest on MDA-MB-231 cells by downregulating the cyclin D1 and cyclin-dependent kinase-6 (Cdk6), as well as upregulating cyclin-dependent kinase inhibitors, p21 and p27 proteins [70]. 

OC also significantly inhibited the proliferation of several human breast cancer MDA-MB-231, MDA-MB-468 cell, MCF-7 cells, BT-474 cells and T-47D cells, with or without induction by hepatocyte growth factor (HGF) or 17β-oestradiol [66,68,69,70,71,78]. OC (20 µM) also significantly reduced the growth of HGF-stimulated MDA-MB-231 3D spheroids [69]. OC-induced apoptosis was more selective against breast cancer cells that highly expressed the mammalian target of rapamycin (mTOR) [71]. This was further illustrated by molecular docking analysis, which revealed that OC possessed high affinity towards the ATP binding pocket of the mTOR homolog, phosphoinositide 3-kinase-γ (PI3K-γ) [71]. In a Z’-LYTE kinase assay from Invitrogen (Carlsbad, CA), OC was shown to inhibit mTOR activity with an IC_50_ value of 708 nM [71]. OC (10 µM) also inhibited the activation of mTOR by reducing its phosphorylation, causing cytotoxicity of MDA-MB-231 cells [71]. 

The antimigratory, anti-invasive and antiproliferative properties of OC were closely related to the inhibition of the HGF receptor which also known as cellular MET (c-MET) tyrosine kinase. Pretreatment of OC abrogated HGF-induced c-MET activation of MDA-MB-231, MCF-7 and BT-474 cells [70]. OC induced the subsequent activation of caspase-8/-3, receptor-interacting protein kinase (RIP) degradation, and PARP cleavage via the downregulation of c-MET signalling in MDA-MB-231 cell apoptosis [70]. Moreover, OC abolished HGF-induced Akt and ERK activation, in a manner similar to the SU11274, c-MET inhibitor [70]. A molecular docking analysis revealed that OC possesses excellent binding affinity towards ATP binding site of c-MET kinase [66,67]. OC was demonstrated to fill in the space between the hinge region and the activation loop of the ATP binding site of c-MET [66,67]. Z’-LYTE c-MET kinase activity assay revealed that OC could inhibit c-MET phosphorylation [66,67,68,69]. In addition, OC-mediated inhibition was effective on wild-type c-MET kinase and mutant c-MET M1250T, but not on mutant c-MET Y1230C and Y1235D [68]. This indicates that OC-mediated c-MET inhibition is required to bind to the c-MET protein in specific orientation. The Omnia^®^ c-MET kinase assay from Life Technologies (Darmstadt, Germany) further confirmed that OC inhibited c-MET kinase via an ATP-competitive binding mechanism [67]. In animal studies, OC (5 mg/kg/day, i.p.) significantly suppressed the tumour growth, proliferation, and angiogenesis of subcutaneous xenografted MDA-MB-231 cells in athymic nude mice [70]. OC also effectively inhibited c-MET activation of MDA-MB-231 xenograft in nude mice [70]. In addition, OC did not significantly change the body weight of nude mice, indicating its low toxicity in animals [70].

Furthermore, structural modifications of OC were performed in order to produce novel and potent OC analogues in anticancer studies [67,68,69]. Busnena et al. (2013) synthesized 10 ester and carbamate OC analogues by substituting the elenoic acid moiety with other functional groups [67]. Among these analogues, tyrosol sinapate (6’-hydroxyphenethyl-3-(7-hydroxyl-6,8-dimethoxyphenyl) acrylate) was demonstrated to have significant c-MET inhibitory activity comparable with OC [67]. A molecular docking analysis suggested that tyrosol sinapate binds to the c-MET ATP pocket [67]. The phenolic sinapate group with a para-positioned hydroxyl group was crucial for c-MET inhibition [67]. In addition, tyrosol sinapate was only slightly cytotoxic to MDA-MB-231 cells with an IC_50_ of 73.7 µM as compared to OC (15 µM) [67]. In a subsequent study, Mohyeldin et al. (2016) synthesized 43 OC analogues for a c-MET inhibition study [68,69]. Homovanillyl sinapate (HVS) (5’-methoxy-6’-hydroxyphenethyl-3-(7-hydroxyl-6,8-dimethoxyphenyl) acrylate) with an additional methoxyl group at the phenethyl moiety compared to tyrosol sinapate, was one of the most potent c-MET inhibitors among these OC analogues [68,69]. HVS was highly selective to breast and prostate cancer cells that highly expressed c-MET [68,69]. In addition, HVS induced antiproliferation, antimigration, and anti-invasion properties in breast and prostate cancer cells with a 4 to 20-fold lower concentration than OC [68,69]. Furthermore, HVS did not cause any significant cytotoxicity in non-tumorigenic MCF-10A cells at up to 80 µM after a 24-h treatment [69]. 

Heat shock protein 90 (Hsp90) are protein chaperones that are important for maintaining the stability, maturation, and signalling of Hsp90 client proteins, like mutated tumour suppressor protein p53, chimeric Bcr-Abl, oestrogen receptor (ER), Cdk4, and Akt [84,85,86,87]. Hsp90 inhibitors like geldanamycin and radicicol have been demonstrated to induce the degradation of these Hsp90 client proteins [88,89]. Some studies have reported that OC can bind to and inhibit heat shock protein 70 (Hsp70) and Hsp90 [79,80]. Surface plasmon resonance (SPR) and molecular docking analysis revealed that OC directly interacted with Hsp90 and easily accommodated into its ATPase active site of Hsp90 protein with an affinity constant 0.87 µM [79]. OC also induced a conformational change in Hsp90, leading to Hsp90 tetrameric structure formation [79]. In addition, OC immobilized with the carbonyl-di-imidazole-agarose beads on its dialdehyde carbonyl moiety was demonstrated to bind to recombinant Hsp90 protein or Hsp90 protein from human histiocytic lymphoma U937 cell lysates [79]. A similar study from Cassiano et al. (2015) also demonstrated that OC (dialdehyde carbonyl moiety) immobilized with 11-azido-3,6,9-trioxaundecan-1-amine-linker-carbonyldiimidazole-agarose beads selectively bound with Hsp70 and Hsp90 [80]. OC might interact with the Hsp90 protein via the 6’-hydroxyphenethyl moiety, because the dialdehyde carbonyl moiety was sacrificed after the agarose bead immobilization procedure. 

In vitro studies have also reported that OC inhibits Hsp90 ATPase activity in a concentration-dependent manner comparable with radicicol (Hsp90 inhibitor) [79]. Similar to radicicol, 24-h OC treatment (30 µM) reduced the expression of Hsp90 client proteins, like Cdk4 and Akt, in U937 cells [79]. However, OC did not upregulate the expression of Hsp70 or Hsp90 proteins, like radicicol [79]. c-MET protein is an example of an Hsp90 client protein [90,91]. However, OC treatment did not induce any significant changes in total c-MET protein expression [70]. Further investigation is needed to verify the inhibitory effects of OC on Hsp70/90. Multiple studies have reported that Hsp90 inhibition could interfere with STAT signalling with minimal to no effect on total STAT protein levels [89,92]. However, STAT3 protein does not require Hsp90 to maintain protein folding and stability [92]. Parallel with these findings, OC was shown to inhibit STAT3 signalling by reducing STAT3 phosphorylation or transcriptional activity without affecting total STAT3 expression [65,72]. To date, no study has been performed to investigate the direct relationship between STAT3 signalling and Hsp90 in OC-mediated apoptosis. 

OC was demonstrated to bind with ER with a 6.7-fold higher binding affinity with ERα than ERβ [81]. OC was shown to be weaker than 17β-oestradiol with a relative ER binding affinity of 0.102% on ERα and 0.0166% to ERβ [81]. In an in vitro study, OC treatment alone was antioestrogenic to ERα-positive MVLN cells and ERβ-positive RNDA cells [82]. OC also significantly suppressed 17β-oestradiol-induced ERα/β activation in MVLN cells and RNDA cells [82]. OC treatment alone also did not alter the ER downstream genes, including *von Willebrand factor*, *ectonucleotide pyrophosphatase/phosphodiesterase* 2 and *aromatase* gene expression in human osteosarcoma U2OS cells that were transfected with both ERα/β [82]. Surprisingly, a high concentration of OC (10 µM) possessed weak oestrogenic activity on U2OS cells transfected with ERα but not ERβ [82]. The authors suggested the discrepancy of their findings might be due to the different cell lines used [82]. 

In addition, a recent study by Ayoub et al. (2017) demonstrated that OC inhibits the 17β-oestradiol-induced proliferation of MCF-7 cells, BT-474 cells, and T-47D cells by interfering with their ER signalling [78]. The combination treatment of OC and tamoxifen (breast cancer chemotherapeutic agent) synergistically suppressed the proliferation of breast cancer cells [78]. OC greatly potentiated the antiproliferative activities of tamoxifen on BT-474 cells with the largest tamoxifen dose reduction index (12.1) [78]. An in silico analysis further revealed that the binding orientation of OC with ER overlapped with the 17β-oestradiol [78]. In addition, the ER-binding site of OC was different to tamoxifen, thus explaining the synergism [78]. OC treatment also reduced the expression of ERα in BT-474 cells [78]. BT-474 cells, but not MCF-7 or T-47D cells, expressed human epidermal growth factor receptor type 2 (HER-2) [78,93]. ER and HER-2 are examples of Hsp90 client proteins. The inhibition of Hsp90 can interfere with ER and HER-2 signalling via protein instability and degradation [86,88,94]. Hence, OC-induced ERα downregulation might be closely related to Hsp90 inhibition, which, in turn, contributes to the synergism between OC and tamoxifen. Results from in vivo studies showed that OC (5 and 10 mg/kg, i.p. three times per week) almost completely suppressed the tumour growth (97% reduction) of subcutaneous mammary gland xenografted BT-474 cells on female athymic nude mice that were pre-implanted with 17β-oestradiol releasing pellets [78]. OC did not significantly change the body weight of the nude mice [78]. The OC-mediated BT-474 xenograft growth suppression involved ERα downregulation [78], thus Hsp90 inhibition was implicated. 

### 4.3. Liver Cancer

Similar to breast cancer studies, OC has also been shown to induce G1 arrest on several human hepatocellular carcinoma cell lines, including Huh-7, HepG2 and HCCLM3 cells [72]. OC at low concentrations (<12.5 µM) inhibited the colony-forming capacities of HepG2 and Hep3B cells [73]. OC also inhibits the migration and invasion of Huh-7 and HepG2 cells [72]. OC attenuates EMT by upregulating E-cadherin and downregulating *N*-cadherin and vimentin [72]. This is mediated by downregulation of the Twist transcription factor but does not involve other transcription factors like Snai1, Zeb1, Slug, and Smad interacting protein-1 (Sip1) [72]. OC also induces cytotoxicity and apoptosis in several human hepatocellular carcinoma cell lines, such as Huh-7, Hep3B, HepG2, HCCLM3 and PLC/PRF/5 cells [72,73], but this action is independent of COX-2 signalling [73]. OC-induced apoptosis of hepatocellular carcinoma cells involves reactive oxygen species (ROS) formation, γ-histone 2Ax (γ-H2Ax) upregulation, p38 phosphorylation, mitochondrial depolarization, caspase-3/7 activation, and PARP cleavage [72,73]. The molecular ordering of OC-induced apoptosis has not been determined; however, the increased production of ROS might serve as the main apoptotic mediator [73]. Pretreatment with N-acetyl cysteine (NAC) was shown to protect HepG2 and Hep3B cells from OC-induced cytotoxicity, apoptosis, PARP cleavage, mitochondrial depolarization and γ-H2Ax upregulation [73]. The ROS produced via mitochondrial respiration complex I and NOX contributed to the apoptosis of hepatocellular carcinoma cells treated with OC [73]. 

STAT3 protein was shown to be highly phosphorylated in primary hepatocellular carcinoma cells isolated from patients [72]. OC-mediated apoptosis and anti-invasion effects are closely related to STAT3 inhibition [72]. Mechanistically, OC inhibits STAT3 signalling via multiple mechanisms [72]. OC inhibits STAT3 signalling via inhibition of STAT3 protein phosphorylation, inhibition on STAT3 nuclear translocation and DNA binding activity, and suppression of STAT3 downstream proteins including cyclin D1, survivin, Bcl-2 and MMP-2 [72]. OC also prevents the binding of the STAT3 protein with the Twist gene promoter [72]. Furthermore, OC was reported to inhibit IL-6-induced STAT3 activation via downregulation of gp80 and gp130 [72]. OC also downregulates STAT3 positive regulator, JAK1/2, and upregulates STAT3 negative regulator SHP-1 expression [72]. OC-mediated STAT3 inhibition does not involve the alteration of Src homology 2 domain tyrosine phosphatase-2 (SHP-2), protein tyrosine phosphatase 1B and Akt expression [72]. The anticancer effects of OC were demonstrated to be dependent on STAT3 signalling because STAT3 overexpression significantly protected HepG2 cells from OC-induced apoptosis and anti-invasion [72]. In addition, OC (5 mg/kg/day; i.p.) also significantly inhibited tumour growth of HCCLM3 and primary hepatocellular carcinoma patient-derived xenograft in male BALB/c athymic nude mice [72]. OC exerted antiproliferation and apoptosis-promoting effects on HCCLM3 and orthotopic hepatocellular carcinoma patient-derived xenograft tumour tissues [72]. OC also significantly reduced the lung metastasis and growth from HCCLM3 xenografts [72]. The molecular mechanism of OC-mediated anticancer activities on HCCLM3 xenografts was in parallel with the in vitro findings [72]. 

### 4.4. Colon Cancer

OC was shown to inhibit the colony-forming capacity and induced cytotoxicity and apoptosis in human colorectal carcinoma HT-29 and SW480 cells [73]. OC-mediated cytotoxicity is selective of HT-29 and HCT116 cells expressing adenosine monophosphate-activated protein kinase (AMPK) [74]. OC was also shown to cause COX-2 downregulation via AMPK activation [74], but OC-mediated cytotoxicity was independent of COX-2 signalling [73]. OC-induced HT29 cells apoptosis is mediated by AMPK-activated p53 protein upregulation and activation [74]. In addition, OC was shown to inhibit AMPK-dependent colony formation capacity in HT-29 cells [74]. Similarly, OC was reported to induce AMPK signalling and inhibit COX-2 expression in normal skin epidermis JB6 Cl41 cells [74]. Subsequently, OC has been shown to cause downstream events of cytotoxicity and apoptosis, including ROS formation, γ-H2Ax upregulation, p38 phosphorylation, mitochondrial depolarization, ATP depletion, downregulation of Bcl-2, caspase-3 activation, PARP cleavage and DNA fragmentation in colorectal carcinoma cells [73,74]. ROS induction mediates OC-induced colorectal carcinoma cell cytotoxicity and apoptosis [73]. In vivo studies have shown that OC significantly reduces tumorigenicity and tumour areas of colorectal carcinoma HT-29 cells in chicken embryos via chorioallantoic membrane assay [74]. 

Interestingly, 48-h OC treatment was not cytotoxic to human colorectal adenocarcinoma Caco-2 cells with IC_50_ value > 150 µM [71]. The researchers suggested that the resistance of Caco-2 cells to OC was possibly due to mTOR signalling and expression [71]. Rapamycin (mTOR complex 1 inhibitor) was not effective against colorectal carcinoma cells [95,96,97]. Therefore, the resistance of Caco-2 cells to OC-mediated cytotoxicity might be partly due to its mTOR complex-2 signalling. Another study, by Abunznait et al. (2011), reported that a 48-h treatment with 5 µM OC increased multidrug efflux transporter P-glycoprotein (P-gp) expression and activity with an EC_50_ of 14 µM in human colon adenocarcinoma LS-180 cells [83]. In other words, the administration of OC might lead to drug resistance in cancer chemotherapy [83]. However, OC-upregulated P-gp expression could be beneficial in Alzheimer disease because P-gp functions as an efflux transporter to remove amyloidβ (Aβ) across the blood–brain barrier [98].

### 4.5. Other Cancer Types

OC was shown to induce cytotoxicity and apoptosis induction at a concentration as low as 7.5 µM in human acute promyelocytic leukaemia HL-60 cells [76]. At a high concentration, OC (120 µM) caused the necrosis of HL-60 cells [76]. In addition, OC was shown to induce cytotoxicity and inhibited proliferation in human myeloma ARH-77 cells and murine myeloma MOPC-31C cells [75]. OC also caused G1 arrest in ARH-77 cells and promoted their apoptosis by the activation of caspase-9/-3 [75]. OC was shown to downregulate MIP-1α mRNA and protein expression in myeloma cells [75], which subsequently led to the suppression of Akt, ERK 1/2 and receptor activator of nuclear factor κB ligand (RANKL) signalling [75]. OC-downregulated Akt signalling was shown to lead to caspase-9 activation [77]. Moreover, OC also activated p38 MAPK signalling due to the suppression of RANKL signalling [75]. About 90% of multiple myeloma patients suffer from bone problems, including bone pain, thinning of bone and osteolytic bone lesions due to upregulated osteoclastic activity [99]. MIP-1α serves as a potent activator for osteoclastogenesis [100,101]. In addition, MIP-1α is overexpressed in multiple myeloma patients but not in normal individuals [102]. Therefore, downregulation of MIP-1α by OC may be beneficial in reducing multiple myeloma-related bone complications.

**Figure 3:**
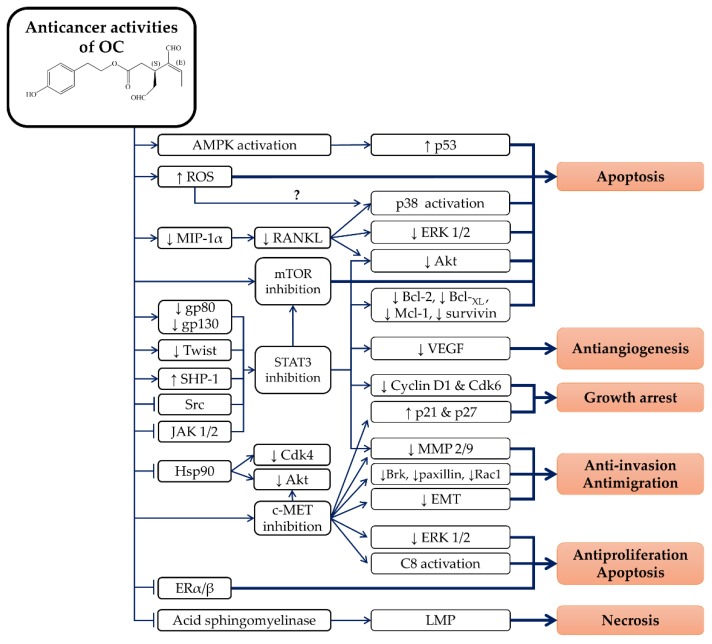
Anticancer properties of OC and the molecular mechanisms of action. Abbreviation: ↓ stands for downregulation; ↑ stands for upregulation; AMPK = adenosine monophosphate-activated protein kinase; ROS = reactive oxygen species; MIP-1α = macrophage inflammatory protein-1α; RANKL = receptor activator of nuclear factor κB ligand; ERK 1/2 = extracellular signal-regulated kinase 1/2; mTOR = mammalian targets of rapamycin; Bcl-2 = B cell lymphoma-2; Bcl-_XL_ = B cell lymphoma-_XL_; Mcl-1 = myeloid cell leukemia-1; gp80 = interleukin-6 receptor; gp130 = interleukin-6 receptor’s signal-transducing subunit; SHP-1 = Src homology 2 domain tyrosine phosphatase-1; JAK 1/2 = Janus kinase 1/2; STAT3 = signal transducer and activator of transcription 3; VEGF = vascular endothelial growth factor; Hsp90 = heat shock protein 90; Cdk4 = cyclin-dependent kinase 4; Cdk6 = cyclin-dependent kinase 6; MMP 2/9 = matrix metalloproteinase 2/9; EMT = epithelial-to-mesenchymal transition; C8 = caspase-8; ERα/β = oestrogen receptor α and β isoform; LMP = lysosomal membrane permeabilization.

In addition, OC has also inhibited migration, invasion, and proliferation of human prostate adenocarcinoma PC-3 cells [66,67,68]. LeGendre et al. (2015) reported that OC (10 µM for 24 h) caused necrosis in PC3, MDA-MB-231, and human pancreas adenocarcinoma BxPC3 cells in a serum-starved condition [77]. OC-induced cancer cell necrosis involves ERK1/2 phosphorylation without the activation of caspase-3 and PARP cleavage [77]. Further investigation revealed that OC was able to inhibit acid sphingomyelinase, which subsequently triggered lysosomal membrane permeabilization (LMP)-mediated necrosis [77]. The upregulation of Hsp70 protein or anionic lipids was shown to protect cells from OC-induced necrosis via lysosomal membrane stabilization [77]. In contrast, OC (10 µM) induced milder cytotoxicity, mainly through apoptosis, in PC3, MDA-MB-231 and BxPC3 cells, with the presence of 10% serum [77]. The anticancer effects of OC are summarized in Figure 3.

## 5. Neuroprotective Effects of OC

OC has shown promising neuroprotective effects in in vitro and in vivo studies. Specifically, OC was able to cross-link and abrogate fibrillization of tau T40 and K18 construct protein via covalent modification in an in vitro assay [103]. The mass spectrometric investigation revealed that OC covalently modified tau K18 protein through Schiff base formation between the ε-amino groups of lysine residues and OC’s aldehyde carbonyl moiety in 1:1 stoichiometry [104]. OC modified tau K18 protein in a temperature- and time-dependent manner [104]. The cross-linking of OC with lysine residues of tau K18 protein formed a stable cyclic pyridinium-like adduct after the rearrangement of the OC dialdehyde carbonyl moiety [104]. Despite the presence of the reactive dialdehyde moiety, OC exerted low or no binding reactivity towards nucleophilic amino acids, such as lysine and arginine [105]. Furthermore, OC was shown to bind to wild-type tau-441 protein as determined by SPR and matrix-assisted laser desorption/ionization mass spectrometry analysis [105]. OC covalently modified the lysine residues of the tau-441 protein [105]. OC induced conformational rearrangement and secondary structural changes of tau-441 from random coil to α-helical moieties [105]. Furthermore, OC was also shown to inhibit arachidonic acid-induced tau protein aggregation into fibrillary structures [105]. 

In addition, OC was also reported to induce amyloid β (Aβ) efflux and clearance in an in vitro model of Alzheimer’s disease [30,52,83]. P-gp and low-density lipoprotein receptor-related protein 1 (LPR1) serve as the efflux transporter to remove Aβ from the brain [30,106,107]. As discussed previously, OC was reported to increase P-gp expression (2.3 fold) and activity (3.4 fold) in LS-180 cells [83]. Abuznait et al. (2013) also reported that OC improves Aβ clearance by upregulating P-gp and low-density lipoprotein receptor-related protein 1 (LRP1) in mouse brain endothelial bEnd3 cells [30]. Chemical inhibition of P-gp and LRP1 prevented OC-mediated Aβ clearance [30]. In addition, OC also enhanced the transport of Aβ across the human brain endothelial hCMEC/D3 cells monolayer through the upregulation of P-gp and LRP1 expression [52]. 

Furthermore, OC also promoted Aβ degradation, reduced astrocytic inflammatory activation and restored the expression of the neuro-supportive protein in astrocyte and neuroblastoma cells [108,109]. Aβ_42_, an Aβ isoform, was reported to be more toxic than Aβ_40_ due to its tendency to aggregate and form toxic Aβ oligomers (Aβo) and fibrils in the pathogenesis of Alzheimer’s disease [109,110]. Batarseh et al. (2017) revealed that Aβo induced astrocyte inflammatory activation and decreased the expression of glutamate transporter-1 (GLT1), glucose transporter-1 (GLUT1), postsynaptic density protein 95 (PSD-95; a postsynaptic marker) and synaptosomal-associated protein 25 (SNAP-25, a presynaptic marker) in human astrocytoma CCF-STTG1 cells and neuroblastoma SH-SY5Y cells transfected with amyloid precursor protein (APP) (SH-SY5Y-APP cells) [109]. Aβo also induced Aβ monomer degradation with the upregulation of the Aβ degradation enzyme, ATP-binding cassette transporter-A1 (ABCA1) [109]. At the same time, Aβo upregulated Aβ production with an increase in the expression of APP, a soluble form of α and β of APP (sαAPP and sβAPP) in SH-SY5Y-APP cells [109]. OC reduced Aβo-mediated astrocyte inflammatory activation by suppressing the expression of GFAP and IL-6 [109]. In addition, OC also restored Aβo-induced downregulation of GLT1, GLUT1, PSD-75 and SNAP-25 level in CCF-STTG1 and SH-SY5Y-APP cells [109]. In addition, OC was shown to alter the structure of Aβo and increase its immunoreactivity [108]. Furthermore, OC was also reported to reduce the synaptopathological effects from toxic Aβ-derived diffusible ligands (ADDLs) by suppressing synaptic deterioration and enhancing the antibody clearance of ADDLs [108]. 

On the other hand, OC did not affect the regulation and accumulation of Aβ influx in the brain as it did not alter the expression and activity of the receptor for advanced glycation end products (RAGE) [30]. OC also did not affect the production of Aβ, whereby the levels of Aβ isoforms (Aβ_40_ and Aβ_42_) and the soluble α and β forms of APP (sαAPP and sβAPP) remained unchanged upon treatment [52]. OC also did not affect Aβo-induced Aβ production in SH-SY5Y-APP cells [109]. In addition, OC did not prevent Aβo-induced Aβ monomer degradation and ABCA1 upregulation [109]. Similarly, OC failed to upregulate LRP1 and Aβ degradation enzymes, including insulin-degrading enzyme (IDE), neprilysin (NEP) and ABCA1 in CCF-STTG1 cells [109]. OC was suggested to act directly on the neuronal cells, but not astrocytes, in exerting its neuroprotective effects [109]. 

**Figure 4:**
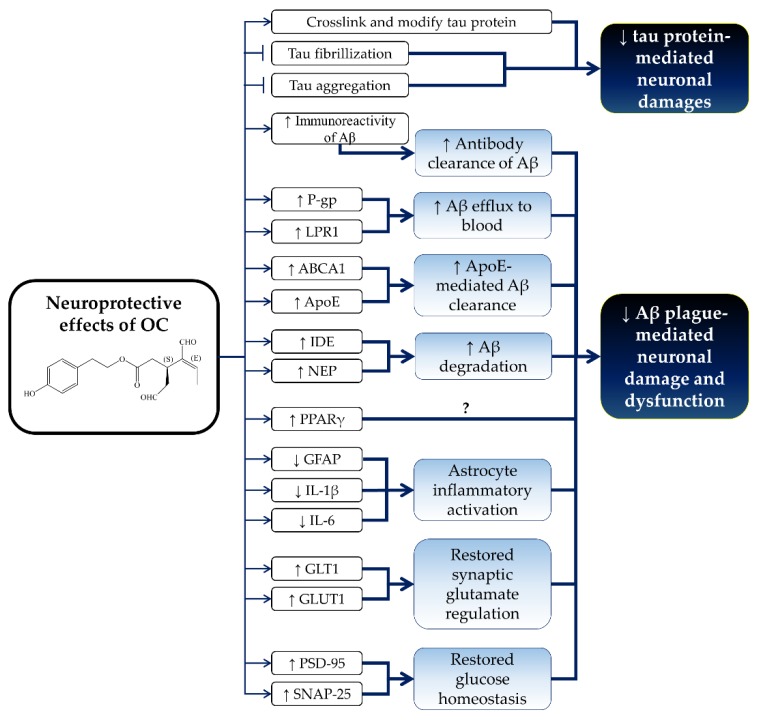
Neuroprotective effects of OC and the molecular mechanisms of action. Abbreviation: ↓ stands for downregulation; ↑ stands for upregulation; Aβ = amyloid β; P-gp = P-glycoprotein; LRP1 = low-density lipoprotein receptor-related protein 1; ABCA1 = ATP-binding cassette transporter-A1; ApoE = apolipoprotein E; IDE = insulin-degrading enzyme; NEP = neprilysin; PPARγ = peroxisome proliferator-activated receptor γ; GFAP = glial fibrillary acidic protein; IL-1β = interleukin-1β; IL-6 = interleukin-6; GLT1 = glutamate transporter-1; GLUT1 = glucose transporter-1; PSD-95 = postsynaptic marker postsynaptic density protein 95; SNAP-25 = synaptosomal-associated protein 25.

There are limited in vivo studies regarding the neuroprotective effects of OC, but results from the available Alzheimer’s disease animal models are promising. Parallel with the in vitro findings, OC reduced astrocytic inflammation and improved the Aβ clearance and degradation in an Aβ-induced Alzheimer’s disease model in mice [30,52]. OC (5 mg/kg/day, i.p.) was reported to reduce astrocytes’ inflammatory activation and decrease GFAP and IL-1β expression in the brains of TgSwDi mice with Alzheimer’s disease [52]. OC was shown to reduce the Aβ load and Aβ-plaque load from the hippocampus of TgSwDI mice [52]. Besides, OC (10 mg/kg, i.p.; twice a day) enhanced clearance of microinjected radioisotope-labelled Aβ_40_ (^125^I-Aβ_40_) in C57BL/6 mice and improved the brain efflux index [30]. OC-mediated Aβ clearance was associated with Aβ efflux by upregulating P-gp and LRP1 in OC-treated mice brain microvessels [30]. In addition, OC increased the degradation of ^125^I-Aβ_40_ via upregulation of Aβ degrading enzyme and insulin-degrading enzyme expression in C57BL/6 mice [30]. OC also enhanced total brain and blood–brain barrier clearances of microinjected ^125^I-Aβ_40_ from the brains of TgSwDI mice via the upregulation of P-gp and LRP1 [52]. Interestingly, OC significantly increased the expression of NEP, IDE, ABCA1 and apolipoprotein E (ApoE) levels in TgSwDI mice, which assisted in ^125^I-Aβ_40_ clearance [52]. Moreover, OC upregulated the nuclear receptor, peroxisome proliferator-activated receptor γ (PPAR γ), but not the liver X receptor or the nuclear receptor retinoid-X receptor in TgSwDI mice [52]. PPARγ levels were found to decrease in the brains of Alzheimer’s disease patients [111]. Furthermore, the activation of PPARγ by PPARγ activator or NSAIDs was demonstrated to suppress Aβ generation via downregulation of β-secretase [112,113]. However, the exact role of OC-mediated PPARγ upregulation in Alzheimer’s disease requires further investigation because OC was previously reported to have no effect on Aβ production [52]. In agreement with the in vitro studies, OC did not affect the expression of APP, sαAPP, sβAPP and total tau protein in TgSwDI mice [52]. OC also did not alter the phosphorylation status of tau protein in Ser214, Ser262, Thr212 and Thr231 [52]. The neuroprotective effects of OC are summarized in Figure 4. 

## 6. Conclusions

OC is an olive phenolic with a strong anti-inflammatory activity. Since many chronic diseases, such as cancers and Alzheimer’s disease, possess an underlying inflammatory origin, OC has the potential to be used as a preventive measure against these conditions. As illustrated by the aforementioned studies, OC exerts its functions through multiple cellular pathways and targets. It could overcome the limitations of monotarget therapy. Most evidence on the health beneficial effects of OC is derived from cellular studies and, to a limited extent, animal studies. Translating the results from cell culture studies to in vivo models or humans is difficult because the concentrations used might not be achievable physiologically. It is also difficult to directly translate the beneficial effects of OC from animal models to humans due to interspecies differences in pharmacokinetics and pharmacodynamics [114,115]. In addition, it is noteworthy that a daily intake of olive oil of 25–50 mL provides < 0.9 mg OC [1,116]. This suggests that the beneficial effects of OC derived from animal studies (5–30 mg/kg) might be difficult to achieve in humans via olive oil intake. The use of pure OC as a supplement is a more likely approach. Therefore, more intensive in vivo or human studies should be encouraged to validate the effects of OC, especially in physiological achievable doses. Since there are many phenolic compounds and fatty acids in olive oil, it could be hypothesized that OC, along with other bioactive components, contributes to the health beneficial effects of olive oil.

## Figures and Tables

**Table 1:** Anticancer properties of OC in various models and their molecular actions.

Model	Molecular Action	Reference
Human melanoma A375 cells and 501Mel cells	Cytotoxicity with downregulation of Bcl-2, Akt, and ERK1/2	[64]
Human melanoma A375 cells and A2058 cells	Cytotoxicity and apoptosis induction with caspase-9/3 activation and PARP cleavage Antimigration and anti-invasion Inhibited STAT3 phosphorylation, nuclear translocation, and transcriptional activity. Downregulated STAT3 downstream protein expression (Mcl-1, Bcl-XL, MMP-2/9, and VEGF)	[65]
HUVEC cells	Antimigration, anti-invasion and antiangiogenesis	[65]
Endothelial colony forming cells	Antiangiogenesis	[66]
Human breast cancer MDA-MB-231 and MCF-7 cells Human prostate cancer PC-3 cells	Antiproliferation, antimigration and anti-invasion with c-MET inhibition	[66]
Human breast cancer MDA-MB-231 cells	Antimigration and anti-invasion with c-MET inhibition	[67]
Human breast cancer MDA-MB-231, MCF-7, and BT-474 cells	Antiproliferation with or without the HGF induction Antiproliferation and G1 cell cycle arrest with downregulation of cyclin D1, Cdk6, Akt and ERK, and upregulation of p21 and p27. Antimigration and anti-invasion via Brk/paxillin/Rac1 suppression Inhibited HGF-induced c-MET activation and EMT. Induced apoptosis with caspase-8/3 activation, PARP cleavage, RIP degradation, and c-MET degradation	[70]
Human breast cancer MDA-MB-231, MCF-7, and T-47D cells	Cytotoxicity and inhibition of mTOR phosphorylation	[71]
Human breast cancer MDA-MB-231, MDA-MB-468, MCF-7, BT-474 and T-47D cells	Anti-proliferation, anti-migration and anti-invasion	[68]
Human breast cancer MDA-MB-231 and MDA-MB-468 cells	Inhibited HGF-induced proliferation, migration and invasion	[69]
Human breast cancer MDA-MB-231 3D spheroids	Inhibited HGF-induced proliferation	[69]
Human breast cancer MCF-7, BT-474, and T-47D cells	Inhibited 17β-oestradiol-induced via ER downregulation Synergistic with tamoxifen-mediated antiproliferation	[78]
Molecular docking analysis	Excellent binding affinity on ATP binding site of c-MET	[66,67]
	High affinity on ATP binding pocket of PI3k-γ	[71]
	Overlapped with the 17β-oestradiol binding site on ER	[78]
Z’-LYTE mTOR kinase assay, Invitrogen, Carlsbad, CA	Inhibited mTOR activity	[71]
Z’-LYTE c-MET kinase assay, Invitrogen, Carlsbad, CA	Inhibited c-MET phosphorylation	[66,67,68,69]
Omnia^®^ c-MET kinase assay, Invitrogen, Carlsbad, CA	Inhibited c-MET in an ATP-competitive manner	[67]
Surface plasmon resonance, molecular docking analysis and in vitro immobilized OC pull-down assay	Interacted and bound with Hsp90	[79]
In vitro immobilized OC pulldown assay	Interacted and bound with Hsp70 and Hsp90	[80]
Human histiocytic lymphoma U937 cells	Downregulated Hsp90 client proteins (Akt and Cdk4)	[79]
PolarScreen^TM^ ERα and ERβ competitor assay, from Life Technologies, Darmstadt, Germany	Bound with ER with relative oestrogen receptor binding affinity 0.102% on ERα and 0.0166% on ERβ	[81]
Human MVLN cells with ERα Human RNDA cells with ERβ	Antioestrogenic activity on ERα/β Inhibited 17β-oestradiol-induced ER activation	[82]
Human osteosarcoma U2OS cells	Weak oestrogenic activity at 10 µM	[82]
Human hepatocellular carcinoma Huh-7, HepG2, and HCCLM3 cells	G1 arrest, antimigration, and anti-invasion Cytotoxicity, and apoptosis induction with caspase-3 activation and PARP cleavage Suppressed EMT via downregulation of Twist transcription factor Inhibited STAT3 signalling and downregulate STAT3 downstream protein expression (cyclin D1, survivin, Bcl-2, and MMP-2) Inhibited IL-6-induced STAT3 activation via downregulation of gp80, gp130, and JAK 1/2, and increased SHP-1 expression.	[72]
Human hepatocellular carcinoma HepG2, Hep3B, Huh-7 and PLC/PRF/5 cells Human colorectal carcinoma HT-29 and SW480 cells	Cytotoxicity and apoptosis with increased ROS formation, γ-H2Ax upregulation, p38 activation, mitochondrial depolarization, caspase-3/7 activity and PARP cleavage. Inhibited colony-forming capacities Produced ROS via mitochondrial respiration complex I and NOX	[73]
Human colon cancer HT-29 and HCT116 cells	Cytotoxicity and apoptosis with AMPK activation, AMPK-mediated p53 activation, ATP depletion, caspase-3 activation, PARP cleavage and DNA fragmentation Inhibited colony formation via AMPK activation	[74]
Human myeloma ARH-77 cells Murine myeloma MOPC-31C cells	Cytotoxicity, antiproliferation, G1 arrest and apoptosis with caspase-9/3 activation Downregulated MIP-1α and led to RANKL, Akt, and ERK1/2 downregulation, but p38 activation	[75]
Human prostate cancer PC-3 cells Human breast cancer MDA-MB-231 cells Human pancreas adenocarcinoma BxPC3 cells	Apoptosis induction Activated ERK 1/2 signalling Inhibited acid sphingomyelinase and caused LMP-mediated necrosis (in the absence of serum)	[77]
Human acute promyelocytic leukaemia HL-60 cells	Cytotoxicity and apoptosis induction	[76]
HT-29 cells inoculation in chorioallantoic membrane assay	Reduced tumour area	[74]
Human colon adenocarcinoma LS-180 cells	Increased P-gp expression and activity	[83]
Human colorectal adenocarcinoma Caco-2 cells Human cervical cancer HeLa cells	Non-cytotoxic with IC_50_ more than 150 µM (48 h)	[71]
Non-tumorigenic human adult dermal fibroblast HDFa cells	Non-cytotoxic up to 60 µM (72 h)	[64]
Non-tumorigenic human mammary epithelial MCF10A cells	Non-cytotoxic up to 60 µM (72 h)	[70]
Non-tumorigenic human liver LO2 cells	Non-cytotoxic up to 80 µM (72 h)	[72]
Murine macrophage J774A.1 cells	Non-cytotoxic up to 100 µM (48 h)	[50]
Isolated primary human hepatocytes	Non-cytotoxic up to 100 µM (72 h)	[73]
Non-tumorigenic human fibroblast BJ cells Rat fibroblast 3Y1 cells Human lung fibroblast IMR90 cells	Non-cytotoxic but cytostatic (72 h) by decreasing retinoblastoma protein phosphorylation Ser608	[77]
Non-tumorigenic mouse epidermis JB6 CI41	Inhibited TPA-induced proliferation and transformation by blocking ERK 1/2 signalling	[74]
Murine chondrocyte ATDC-5 cells	Cytotoxic at high concentration (25 µM) with p38 activation	[51]
Subcutaneous A375 xenograft on male BALB/c athymic nude mice	Inhibited tumour growth, proliferation and angiogenesis with STAT3, Src kinase and JAK2 inhibition.	[65]
Injected A375 xenograft on the tail vein of male BALB/c athymic nude mice	Suppressed lung metastatic nodules number and growth	[65]
Mammary gland subcutaneous MDA-MB-231 xenograft in female athymic nude mice	Inhibited tumour growth, proliferation, and angiogenesis without significant changes in mean body weight	[70]
Mammary gland subcutaneous BT-474 xenograft in female athymic nude mice with 17β-oestradiol releasing pellets preimplantation	Inhibited tumour growth and downregulate ERα without affecting body weight	[78]
HCCLM3 xenograft in male BALB/c athymic nude mice	Induced apoptosis and inhibit tumour growth and proliferation Suppressed lung metastasis from tail vein injection of xenograft	[72]
Hepatocellular carcinoma patient-derived xenograft in the liver of male BALB/c athymic nude mice	Inhibited tumour growth and proliferation	[72]

Abbreviation: Bcl-2 = B cell lymphoma-2; ERK = extracellular signal-regulated kinase; PARP = poly (adenosine diphosphate-ribose) polymerase; STAT3 = signal transducer and activator of transcription 3; Mcl-1 = myeloid cell leukemia-1; Bcl-_XL_ = B cell lymphoma-_XL_; MMP-2/9 = matrix metalloproteinase 2/9; VEGF = vascular endothelial growth factor; HUVEC cells = human umbilical vascular endothelial cells; HGF = hepatocyte growth factor; c-MET = HGF receptor or cellular MET tyrosine kinase; Cdk4 = cyclin-dependent kinase 4; Cdk6 = cyclin-dependent kinase 6; Brk = breast tumour kinase; EMT = epithelial-to-mesenchymal transition; RIP = receptor-interacting protein kinase; mTOR = mammalian targets of rapamycin; ER = oestrogen receptor; ERα = oestrogen receptor α isoform; ERβ = oestrogen receptor β isoform; MVLN cells = ERα-positive cell line derived from MCF-7 cells that has been stably transfected with a vitellogenin-2 promoter/firefly luciferase reporter construct; RNDA cells = rat brain raphe nuclei-derived RN46-A-B14 cells that stably transfected with transgenic human ERβ; PI3k-γ = phosphoinositide 3-kinase-γ; ATP = adenosine triphosphate; Hsp70 = heat shock protein 70; Hsp90 = heat shock protein 90; IL-6 = interleukin-6; gp80 = IL-6 receptor; gp130 = IL-6 receptor’s signal-transducing subunit; JAK 1/2 = Janus kinase 1/2; SHP-1 = Src homology 2 domain tyrosine phosphatase-1; ROS = reactive oxygen species; γ-H2Ax = γ-histone 2Ax; NOX = nicotinamide adenine dinucleotide phosphate oxidase; AMPK = adenosine monophosphate-activated protein kinase; MIP-1α = macrophage inflammatory protein-1α; RANKL = receptor activator of nuclear factor κB ligand; LMP = lysosomal membrane permeabilization; P-gp = P-glycoprotein; TPA = 12-*O*-tetradecanoylphorbol-13-acetate.
